# 

**Published:** 1888-09-22

**Authors:** 


### CONTENTS.

**Constructive Charity** - - - - - - - 397

**Vanity or Madness?** - - - - - - - 398

**Benighted** - - - - - - - - 398

**The Doctor's Pulpit.**—Middle Age - - - - 399

**The Hospitals Association and Patients' Contributions.**—Speech by Mr. Henry C. Burdett - - - 400

**The National Pension Fund** - - - - 401

**In and Out Among the Hospitals.** By a Secretary of Ten Years' Standing - - - - - - 402

**Notes and News** - - - - - - - 404

**Hints for the Sick-room.** By M. M. Basil, M.A., M.B.—Administration of Medicines - - - - - 403

**Everybody's Page** - - - - - - - 406

**Annotations.**—Fresh Fruit as Food.—The Battle of the Diets - 407

**The Doctor in the West-end Mews.** By E. Lloyd Jones, M.B., etc. - - - - - - - 408

**An Ishmaelite.** By Leslie Keith, Author of "The Chilcotes," "East and West," etc. - - - - - - 409

**An Asylum Romance.** Being the Tale of a Traveller - 411

**The Children's Corner** - - - - - - 412

### EXTRA SUPPLEMENT.—THE NURSING MIRROR.

En Passant.—Our List of Vacancies.—The St. John Sisterhood.—The Whitechapel Murder.—An American Nurse's Book.—The National Pension Fund for Nurses.—Strange Death of a Clergyman.—Strange Advertisements.—Courage - - xcvii
Original Articles.—A Sister's Holiday. Part II. - - - xcvii
Everybody's Opinion.—The Question of Courage.—Nursing and Emotion - - - - - - - - - xcix
Anecdotes of Personal Adventure - - - - - xcix
Appointments.—Vacancies.—Notes and Queries - - - xcix
Nursing and Fees.—Private Nursing - - - - xcix

